# The cryoEM structure of cytochrome *bd* from *C. glutamicum* provides novel insights into structural properties of actinobacterial terminal oxidases

**DOI:** 10.3389/fchem.2022.1085463

**Published:** 2023-01-04

**Authors:** Tamara N. Grund, Yoshiki Kabashima, Tomoichirou Kusumoto, Di Wu, Sonja Welsch, Junshi Sakamoto, Hartmut Michel, Schara Safarian

**Affiliations:** ^1^ Department of Molecular Membrane Biology, Max Planck Institute of Biophysics, Frankfurt, Germany; ^2^ Department of Bioscience and Bioinformatics, Kyushu Institute of Technology, Fukuoka, Japan; ^3^ Central Electron Microscopy Facility, Max Planck Institute of Biophysics, Frankfurt, Germany; ^4^ Department of Microbiology and Immunology, School of Biomedical Sciences, University of Otago, Dunedin, New Zealand; ^5^ Fraunhofer Institute for Translational Medicine and Pharmacology ITMP Frankfurt, Frankfurt, Germany

**Keywords:** CryoEM, microbiology, oxidases, proton channel, electrochemistry

## Abstract

Cytochromes *bd* are essential for microaerobic respiration of many prokaryotes including a number of human pathogens. These enzymes catalyze the reduction of molecular oxygen to water using quinols as electron donors. Their importance for prokaryotic survival and the absence of eukaryotic homologs make these enzyme ideal targets for antimicrobial drugs. Here, we determined the cryoEM structure of the menaquinol-oxidizing cytochrome *bd*-type oxygen reductase of the facultative anaerobic Actinobacterium *Corynebacterium glutamicum* at a resolution of 2.7 Å. The obtained structure adopts the signature pseudosymmetrical heterodimeric architecture of canonical cytochrome *bd* oxidases formed by the core subunits CydA and CydB. No accessory subunits were identified for this cytochrome *bd* homolog. The two *b*-type hemes and the oxygen binding heme *d* are organized in a triangular geometry with a protein environment around these redox cofactors similar to that of the closely related cytochrome *bd* from *M. tuberculosis*. We identified oxygen and a proton conducting channels emerging from the membrane space and the cytoplasm, respectively. Compared to the prototypical enzyme homolog from the *E. coli*, the most apparent difference is found in the location and size of the proton channel entry site. In canonical cytochrome *bd* oxidases quinol oxidation occurs at the highly flexible periplasmic Q-loop located in the loop region between TMHs six and seven. An alternative quinol-binding site near heme *b*_595_ was previously identified for cytochrome *bd* from *M. tuberculosis*. We discuss the relevance of the two quinol oxidation sites in actinobacterial *bd*-type oxidases and highlight important differences that may explain functional and electrochemical differences between *C. glutamicum* and *M. tuberculosis*. This study expands our current understanding of the structural diversity of actinobacterial and proteobacterial cytochrome *bd* oxygen reductases and provides deeper insights into the unique structural and functional properties of various cytochrome *bd* variants from different phylae.

## Introduction

An estimated 2.4 billion years ago, the “Great Oxidation Event”, presumably originating from the activity of the photosynthetic ancestors of cyanobacteria, raised atmospheric O_2_ levels over four orders of magnitude (to about 10% of current levels) and drastically changed the evolutionary course of life while also leading to one of the greatest mass extinction events on earth (Schirrmeister et al., 2013; Lyons et al., 2014). Certain organisms managed to escape oxidative damage by inhabiting oxygen-free niches. However, the more important adaption to these extreme environmental conditions was the evolution of metalloproteins that catalyze the reduction of highly toxic dioxygen to harmless water and at the same time harness the oxidizing power of oxygen for organismal energy metabolism (Hoganson et al., 1998).

Among these enzymes, terminal oxygen reductases catalyze the reduction of molecular oxygen to water employing electrons from the oxidation of either cytochrome *c* (cyt. *c*) or membrane dissolved quinols (QH_2_). They are classified into i) heme-copper oxidases (HCO), which contain, as a unifying characteristic, a heme-copper binuclear center, ii) alternative oxidases (AOX), first identified in plants, and iii) cytochrome *bd* oxidases, which are only present in prokaryotes (May et al., 2017; Borisov and Siletsky, 2019). All so-far experimentally characterized *bd*-type oxidases use exclusively quinols as substrates, whereas HCOs comprise both cyt. *c*- and QH_2_-oxidizing members.

The cytochrome *bd* enzyme was first described nearly a century ago and since then has been identified as a membrane-integrated respiratory terminal oxidase unique to the prokaryotic domain, including a number of human pathogens (Cook and Poole, 2016; Borisov et al., 2020). While *bd*-type enzymes catalyze the reduction of molecular oxygen to water, they do not actively pump protons across the cytoplasmic membrane as do members of the HCO superfamily. Instead, cytochrome *bd* generates an electrochemical proton gradient *via* charge separation by consuming substrate protons from the cytoplasmic space and the release of protons upon quinol oxidation to the perisplasm. Thus, the contribution to the *proton motive force* (*pmf*) is smaller than that of proton-pumping HCO-type oxidases (Puustinen et al., 1991; Borisov et al., 2011; Borisov and Verkhovsky, 2015).

Canonical cytochrome *bd* oxidases share a common core architecture of two subunits, denoted CydA and CydB, which may be accompanied by up to two additional single-transmembrane helix subunits (Miller and Gennis, 1983; VanOrsdel et al., 2013; Hoeser et al., 2014; Safarian et al., 2016; Safarian et al., 2019; Theßeling et al., 2019; Grund et al., 2021; Safarian et al., 2021; Wang et al., 2021; Friedrich et al., 2022). As implied by designation, cytochromes *bd* contain two *b*-type hemes (low-spin *b*_558_, high-spin *b*_595_) and one *d*-type heme involved in quinol oxidation, electron transfer and oxygen reduction. Substrate-binding occurs within CydA at the quinol binding domain (Q-loop) located on the periplasmic side of the membrane. Members of the canonical cytochrome *bd* oxidases are further subdivided into those containing either a short or a long hydrophilic Q-loop domain (S- and L-subfamily, respectively) (Osborne and Gennis, 1999; Sakamoto et al., 1999; Kusumoto et al., 2000). The role of the N-terminal insertion of the Q-loop domain has been studied previously, yet it still remains elusive whether this extension fulfils a purely structural or additionally also a functional role within cytochrome *bd* oxidases (Goojani et al., 2020; Theßeling et al., 2020).

Cytochromes *bd* are characterized by high oxygen affinity, as well as their resistance to cyanide inhibition (IC_50_ concentration for KCN of cytochrome *bo*_3_ from *E. coli*: 10 μM; IC_50_ concentration for KCN of cytochrome *bd*-I from *E. coli*: 2 mM) (Kita et al., 1984; Borisov and Verkhovsky, 2015). Extreme cyanide insensitivity as well as an apparent lack of a heme *d* signal, led to the sub-classification of cytochromes *bd* into the non-canonical cyanide-insensitive quinol oxidases (CIO) and canonical *bd*-type enzymes (Borisov et al., 2011). In CIOs, heme *d* is replaced by a second high-spin *b*-type heme (Azarkina et al., 1999; Jackson et al., 2007; Mogi et al., 2009). CIO-type terminal oxidases are encoded by the *cio* operon. The deduced amino acid sequences of CioA and CioB are homologous to CydA and CydB (Cunningham et al., 1997; Quesada et al., 2007). CIOs are found *inter alia* in *Pseudomonas aeruginosa* and *Pseudomonas pseudoalcaligenes*, and are assumed to enable aerobic respiration under cyanogenic and microaerobic growth conditions. This is of relevance for the opportunistic pathogen *P. aeruginosa* to attain full pathogenicity in the cyanide-mediated paralytic killing of nematodes (Cunningham et al., 1997; Quesada et al., 2007).

We previously reported X-ray and cryoEM structures of cytochrome *bd* oxidases from *Geobacillus thermodenitrificans* (3.8 Å), *Escherichia coli* (2.7 Å *bd*-I, 2.1 Å *bd*-II), and *Mycobacterium tuberculosis* (2.6 Å) (Safarian et al., 2016; Safarian et al., 2019; Grund et al., 2021; Safarian et al., 2021). While these structures show a common core architecture, cofactor arrangements, accessory subunit compositions, and Q-loop architectures indicate structural and functional diversity within the cytochrome *bd* family.

Here, we determined the cryoEM structure of the actinobacterial cytochrome *bd* from *Corynebacterium glutamicum* to 2.7 Å which is closely related to the mycobacterial cytochrome *bd* oxidases and at the same time displays spectroscopic and electrochemical properties closely matching those of cytochrome *bd*-I from *E. coli* (Nikolaev et al., 2021).

## Results

The menaquinol oxidizing cytochrome *bd*-type oxygen reductase of the facultative anaerobic Actinobacterium *C. glutamicum* (*Cgbd*) was produced under the activity of its native promoter without an affinity tag as described previously (Kabashima et al., 2009) (Supplementary Figure S1). The structure of the natively purified oxidase was determined *via* single-particle analysis cryoEM. A total of 13,390 movies were collected on a Titan Krios G3i microscope equipped with a Gatan K3 camera. The final map refined to 2.7 Å resolution (Supplementary Figures S2–S4, Supplementary Table S1).

**FIGURE 1:**
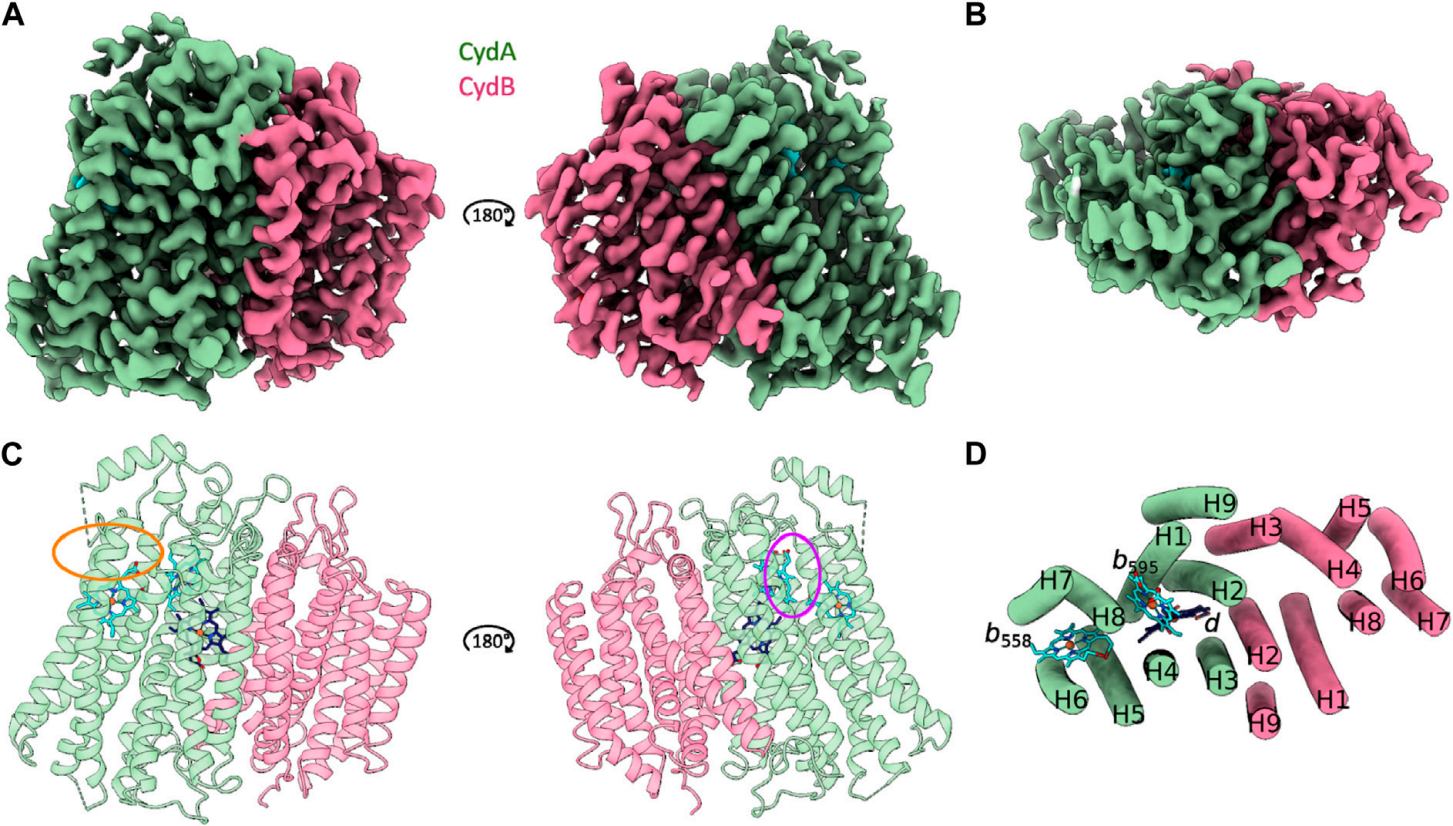
CryoEM structure of *C. glutamicum* cytochrome *bd* at 2.7 Å resolution. **(A and C)** Side view representations of the cryoEM map density and its corresponding ribbon model of the heterodimeric *C. glutamicum* cytochrome *bd* structure. The substrate quinol binding site 1 at the Q-loop in proximity to heme *b*_558_ is highlighted in orange. The second putative quinol binding site next to heme *b*_595_ is shown in purple. **(B and D)** Top view representation of the cryoEM map density and the corresponding tube model of the *Cgbd* structure highlighting the pseudosymmetrical arrangement of the two four helix bundles and the additional peripheral helix per subunit. Color code: CydA, green; CydB, pink; *b*-type hemes, cyan; heme *d*, dark blue.

Cytochrome *bd* from *C. glutamicum* is characterized by the canonical cytochrome *bd* architecture comprising the larger CydA and the slightly smaller CydB subunits. Each subunit is composed of two four-helix bundle motifs and an additional peripheral helix. The heterodimeric *Cgbd* forms a pseudosymmetrical complex with C2 symmetry (Figure 1). The dimerization interface is composed of the symmetry related TMHs 2, 3, and 9 of CydA and CydB, respectively. Subunit interactions are mostly characterized by van-der-Waals interactions. Unlike the *E. coli* and *G. thermodenitrificans* cytochrome *bd* oxidases, *Cgbd* does not contain small accessory subunits which resembles the composition of the closely related mycobacterial oxidases (Safarian et al., 2021; Wang et al., 2021). *C. glutamicum* and *M. tuberculosis* both belong to the phylum of Actinobacteria. The amino acid sequences of their core subunits share a sequence identity of 62% and 48% for CydA and CydB, respectively.

The catalytically active subunit CydA of *Cgbd* harbors three prosthetic heme groups (*b*_558_, *b*_595_, and *d*) and contains the quinol oxidizing domain (Q-loop). The Q-loop is located between TMHs six and seven and protrudes into the periplasm. While the Q_C_ region is fully resolved, residues 268 to 317 which constitute the Q_N_ region could not be traced with high confidence. The Q-loop starts with the short helix *Qh*1 comprising the conserved residues Lys^263.A^_*Cgbd*_ and Glu^268.A^_*Cgbd*_. These residues have been implicated in quinol binding and oxidation as well as coordination of propionate A from heme *b*_558_ in cytochromes *bd* from Proteobacteria (Matsumoto et al., 2006; Mogi et al., 2006). The Q_N_ region of *Cgbd* appears to be characterized by increased movement dynamics similar to what has been observed for cytochrome *bd* from *Mycobacterium smegmatis* (*Msbd*) (Wang et al., 2021). The Q_C_ region of *Cgbd* is fully resolved and consists of the periplasm exposed helix *Qh*3 that is followed by a 180° turn and leads back to TMH7. In the cytochrome *bd* structure of *Mycobacterium tuberculosis* (*Mtbd*), a hydrophobic cluster stabilizing the interaction of *Qh*3 with the periplasmic loop connecting TMHs eight and nine (PL8) was identified (Safarian et al., 2021). The residues Pro^409.A^_*Cgbd*_, Trp^410.A^_*Cgbd*_, and Pro^414.A^_*Cgbd*_ of PL8 and Tyr^335.A^_*Cgbd*_ of *Qh*3 are conserved in *Cgbd*, while the residues Tyr^321.A^_*Mtbd*_ and Phe^325.A^_*Mtbd*_ of *Qh*3 are replaced by Ala^326.A^_*Cgbd*_ and Tyr^330.A^_*Cgbd*_. Since the residues Tyr^335.A^_*Cgbd*_ and Tyr^330.A^_*Cgbd*_, which are relevant for the activity of the mycobacterial enzyme are conserved, a common role of *Qh*3 and periplasmic loop 8 (PL8) in stabilization can be inferred (Sviriaeva et al., 2020).

**FIGURE 2:**
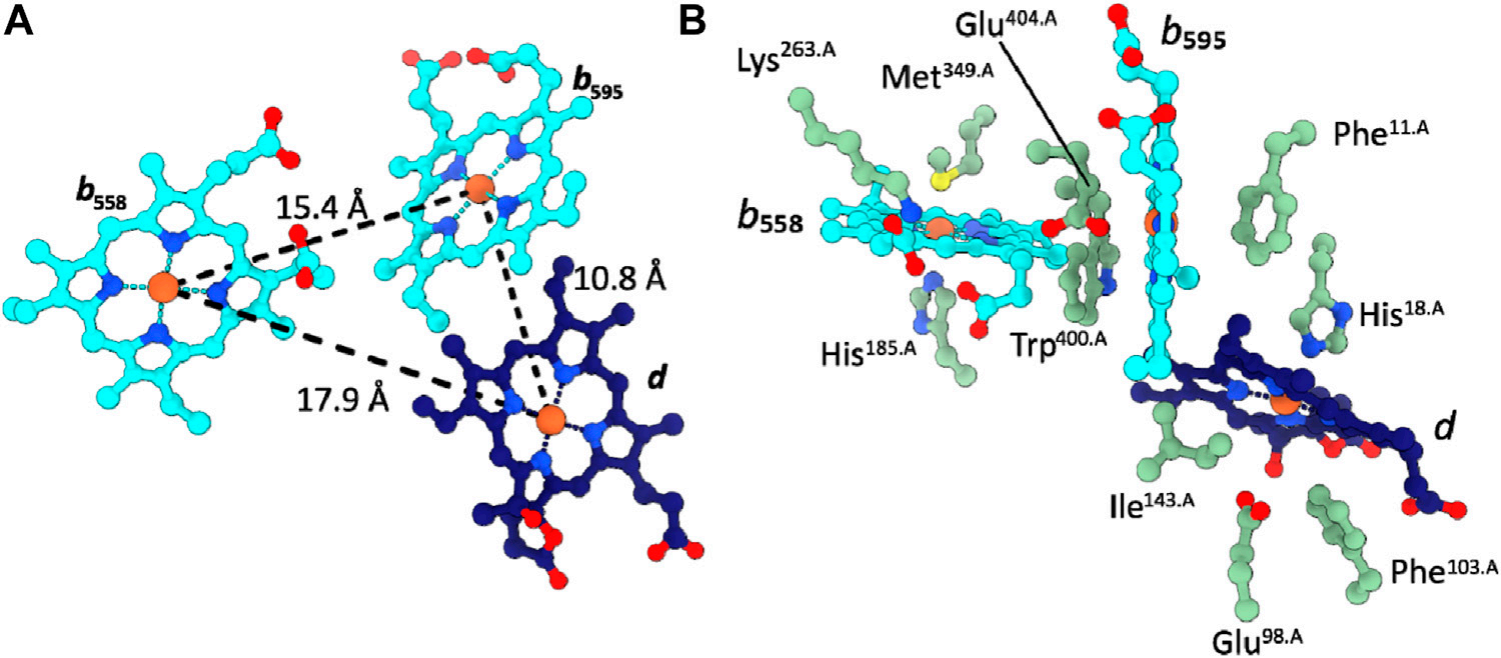
Cofactor arrangement in *Cgbd*. **(A)** Triangular heme arrangement in CydA. **(B)** Axial ligands and proximal side chains of the prosthetic heme groups in *Cgbd*. Color code: CydA, green; *b*-type hemes, cyan; heme *d*, dark blue.

The two *b*-type hemes *b*_558_ and *b*_595_ as well as the chlorin-type heme *d* are organized in the well-characterized triangular cytochrome *bd-*specific geometry and are located in proximity of the periplasmic surface (Figure 2). The coordinating and vicinal residues, as well as the arrangement of the *b*-type hemes and heme *d* are conserved between cytochromes *bd* from Proteobacteria and Actinobacteria (Safarian et al., 2019; Theßeling et al., 2019; Grauel et al., 2021; Grund et al., 2021; Safarian et al., 2021; Wang et al., 2021). A noteable variation in the triangular heme arrangement is however found in the structure of cytochrome *bd* from *Geobacillus thermodenitrificans* (*Gtbd*), in which the high-spin heme *b* and heme *d* have “switched” positions.

The structural subunit CydB does not harbor cofactors or structural quinone molecules as observed in the proteobacterial cytochromes *bd*-I and *bd*-II (Safarian et al., 2019; Theßeling et al., 2019; Grauel et al., 2021; Grund et al., 2021). This is in agreement with the structures of the closely related *Mtbd* and *Msbd* that also lack structural quinones in the smaller subunit CydB (Safarian et al., 2021; Wang et al., 2021). Analogous to *Mtbd*, TMHs six and seven in CydB are connected by a short linker of three amino acids (CydB_*Cgbd*_: amino acids 220–222), whereas the proteobacterial enzymes contain an elongated β-sheet extending along the periplasmic surface of CydB and a short α-helix oriented in a roughly 90° angle to TMH6 (Supplementary Figure S5).

**FIGURE 3:**
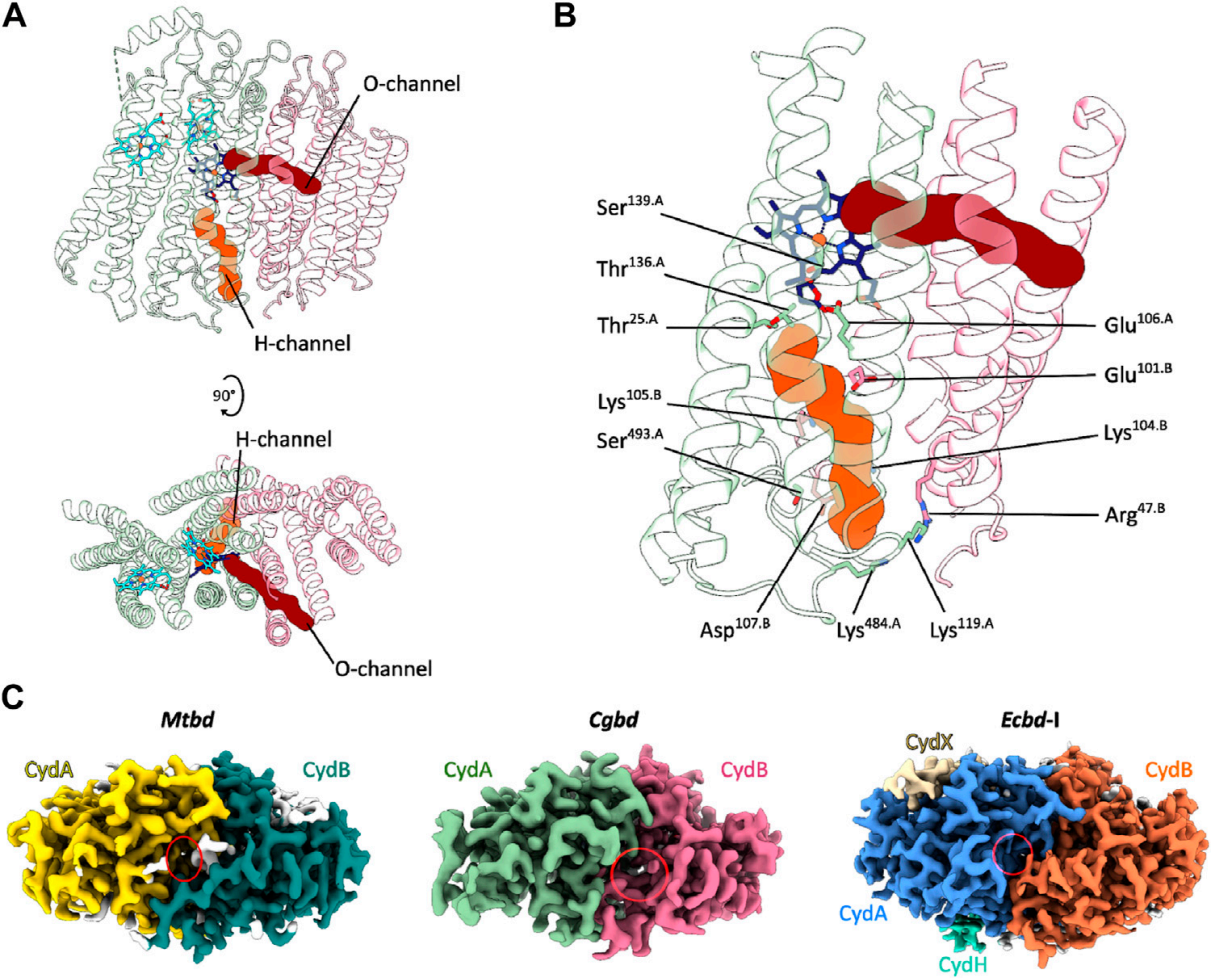
Oxygen and proton pathways in *Cgbd*. **(A)** Oxygen (O) and proton (H) channels predicted by MOLE2. The O-channel starts at the membrane interface between TMHs one, nine and two of CydB and TMH three of CydA and continues towards heme *d*. The predicted H-channel allows proton transfer from the cytoplasm to the reduction site. **(B)** Hydrophilic residues lining the H-channel. **(C)** Variation in location and size of the cytoplasmic proton channel entry sites in cytochromes *bd*. Depicted are the cryoEM maps of *Mtbd* (EMD-12451) (Safarian et al., 2021), *Cgbd* and *Ecbd*-I (EMD-4908) (Safarian et al., 2019) viewed from the cytoplasmic side of the membrane. The cavity elongating into the H-channel is highlighted by a red ellipse.

The structural framework of a cytochrome *bd*-type enzyme requires, in addition to domains and cofactors for substrate oxidation and electron transfer, specific channels and cavities for allowing the access of protons and dioxygen to the reduction site at heme *d*, which is buried deeply within the core of the enzyme at the interface of CydA and CydB. In the *Cgbd* structure, both an oxygen and a proton pathway were identified (Figures 3A, B) (Sehnal et al., 2013; Pravda et al., 2018). The proton channel (H-channel) runs perpendicular to the membrane plane and connects the cytoplasm to the active site at heme *d*. The H-channel is predicted to start as a shallow cavity at the cytoplasmic site in proximity to TMHs two and three of CydB and subsequently constricts and elongates at the interface of CydA and CydB towards the reduction site. The entrance of the H-channel is shifted towards CydB compared to the proteobacterial channels (Figure 3C). The typical opening in the proteobacterial structures is obstructed by the extended C-terminal stretch of CydA in *Cgbd*, while the entrance of the H-channel of *Cgbd* is blocked by the short cytoplasmic C-terminal helix of CydB/AppB in cytochromes *bd* of *E. coli*. On the other hand, the location of the hydrophobic oxygen channel (O-channel) is matching well with the pathways observed in the proteobacterial and mycobacterial enzymes. The O-channel is predominantly lined by hydrophobic and uncharged residues and starts at the lipid interface between TMHs one and nine of CydB and extends parallel to the membrane plane, connecting the lipid bilayer to the reduction site at the chlorin-type heme *d*.

## Discussion

Two substrate quinone binding sites have been characterized for *bd*-type oxidases (for a comparison of all quinone sites please see Supplementary Figure S6). One at the Q-loop next to heme *b*_558_ which is best characterised in *Ecbd*s (site 1)*.* A second quinol binding-site composed of TMH1, TMH9 and heme *b*_595_ has been identified exclusively in *Mtbd* (site 2) (Safarian et al., 2019; Grauel et al., 2021; Safarian et al., 2021). At first glance, the overall structure of *Cgbd* is showing strong resemblance to that of *Mtbd*, however the structure of Q*h*3-PL8 region near site 1 is slightly different.

**FIGURE 4:**
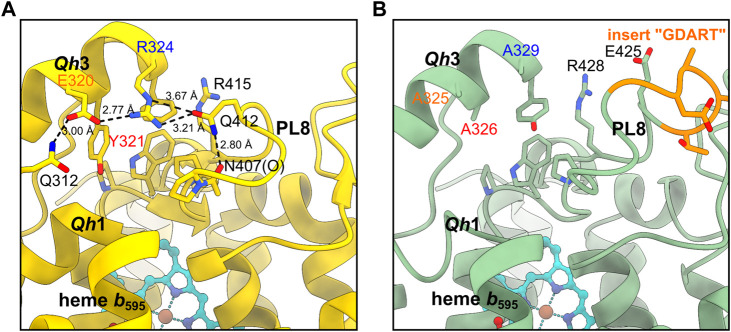
Comparison of the Qh3-PL8 region in **(A)** *Mtbd* and **(B)** *Cgbd*. The stabilizing residues E320, Y321 and R324 of Q*h*3 in *Mtbd* (7NKZ) (Safarian et al., 2021) are replaced by alanines in *Cgbd*, presumably leading to a weaker interaction between Q*h*3 and PL8.

In *Mtbd*, the Q*h*3-PL8 region is mostly stabilized by a hydrophobic cluster composed of aromatic side chains as described previously (Safarian et al., 2021). In addition, a hydrogen bond between the side chain carbonyl group of Gln^412.A^_*Mtbd*_ and Arg^324.A^_*Mtbd*_ seems to strengthen the interaction between PL8 and Q*h*3 (Figure 4). Furthermore, electrostatic interactions between Arg^324.A^, Glu^320.A^, and Gln^312.A^ seem to rigidify the conformation of the *Mtbd* Qc region even more. As Glu^320.A^_*Mtbd*_, Tyr^321.A^_*Mtbd*_ and Arg^324.A^_*Mtbd*_ located within Qh3 are all substituted to alanine residues in homologous positions of *Cgbd* (Ala^325.A^_*Cgbd*_, Ala^326.A^_*Cgbd*_ and Ala^329.A^_*Cgbd*_), it is conceivable that the Q*h*3-PL8 interaction in *Cgbd* might be weaker than in *Mtbd*. In consequence, the local charge and steric environment in close proximity of the well-conserved Gln^260.A^_*Cgbd*_ and Lys^263.A^_*Cgbd*_ residues of Q*h*1, which have been suggested to be important for quinol oxidation in *Ecbd*-I, is significantly different (Mogi et al., 2006). These structural dissimilarities may be first indications that site 1 could represent the quinol binding and oxidation region in *Cgbd.* The increased movement of the *Cgbd* Q_N_ region reflected by unclearly-resolved densities of residues 268 to 317 (CydA) may support a model in which site 1 represents the electrochemically active substrate-binding domain. In fact, quinol oxidase activity of *Cgbd* is about 200–400 s^−1^ (Kusumoto et al., 2000), considerably higher than that of *Msbd* (22 s^−1^) (Wang et al., 2021).

In contrast to the structures of *Cgbd* and *Ecbd-*I*,* the Q-loop domain is fully resolved in *Mtbd* (Safarian et al., 2021)*.* This may be because the Q_N_ region of *Mtbd* contains an internal disulfide bond which contributes to an overall increased stability and rigidity. Based on this unique finding, we previously suggested that the existence or absence of a disulfide bond within the Q-loop domain may correlate with utilization of either site 1 or site 2 as the electron entry route from substrate quinols (Safarian et al., 2021). Interestingly, these two cysteine residues (Cys^271.A^_*Cgbd*_ and Cys^290.A^_*Cgbd*_) are conserved in *Cgbd,* yet our structure suggests that they are not forming a covalent bond, furthermore raising the question about the mechanism of disulfide bond generation and breakage within Q-loops of cytochromes *bd* in a physiological and metabolic context.

**FIGURE 5:**
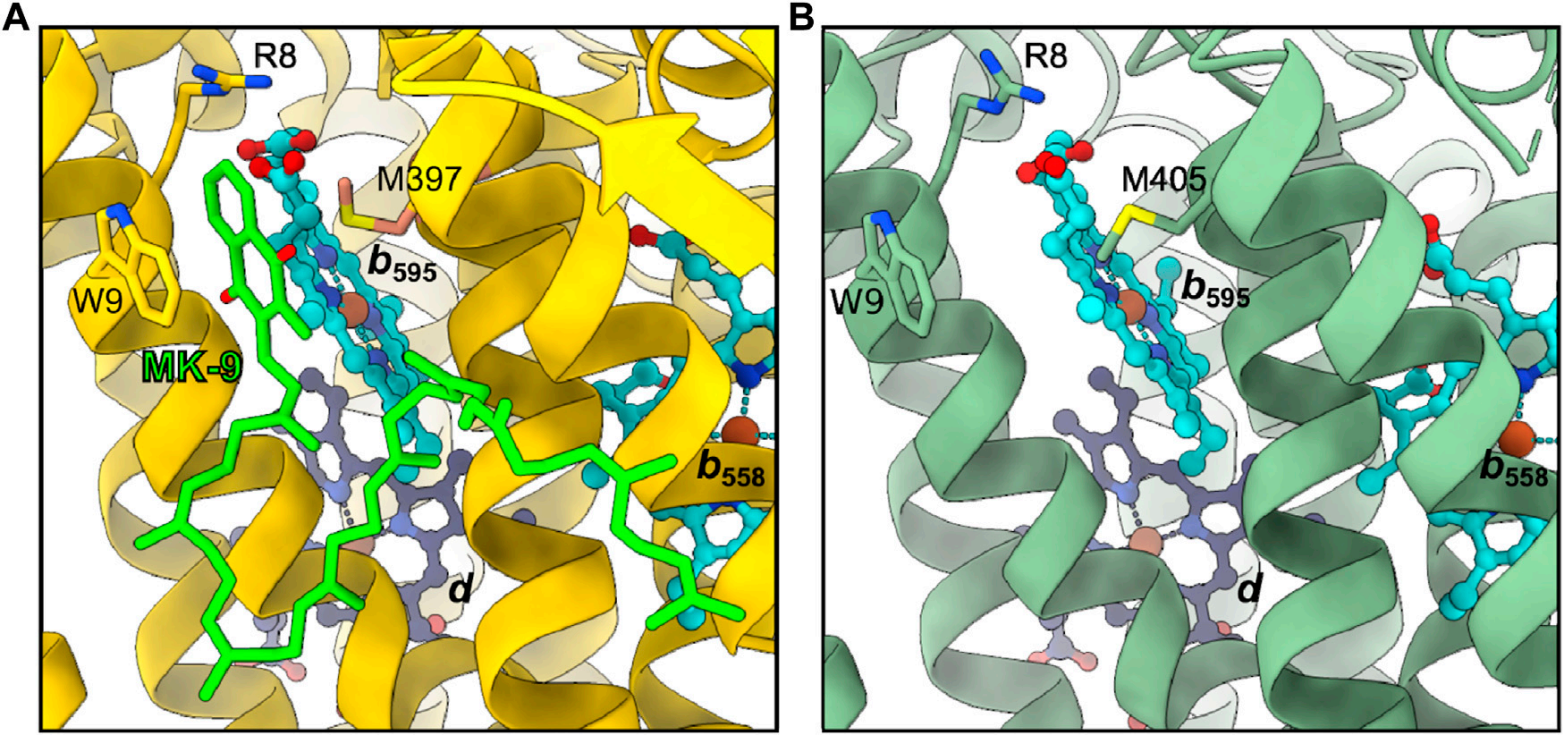
Second quinol binding site. **(A)** In *Mtbd*, site 2 harbors a bound MK-9 molecule stabilized by W9, R8 and M397. **(B)** Site 2 appears unoccupied in *Cgbd*, however the residues interacting with MK-9 in *Mtbd* are conserved in *Cgbd*.

In *Mtbd*, site 2 is occupied by a MK-9 group, while it is appearing empty in *Cgbd* (Figure 5) (Safarian et al., 2021). At higher contour levels a weak density might be present, yet as judged by its size and shape at the current resolution, it more likely represents a bound lipid molecule rather than a quinone group (Supplementary Figure S3B). Nevertheless, the local environment formed by TMH1, TMH9 and heme *b*_595_ in *Cgbd* is almost identical to that of *Mtbd*. The residues forming the binding pocket for the naphthoquinone head group of MK-9 in *Mtbd* are conserved in *Cgbd* (Arg^8.A^_*Cgbd*_, Trp^9.A^_*Cgbd*_ and Met^405.A^_*Cgbd*_), and thus site 2 may still represent a presumable secondary MK-9 biding site. In *Ecbd*-I, site 2 is occupied by the accessory subunit CydH, which forms a different surface at this peripheral site of the cytochrome *bd* complex and consequently does not form any cavities which would allow for quinol binding close to the heme *b*_595_ group. The absence of the CydH subunit and the resulting access to site 2 might furthermore be the reason why we could previously show that the addition of menaquinones modulates the activity of *Ecbd*-II in a concentration dependent manner (Grund et al., 2021).

We have previously reported that preincubation of *Cgbd* and *Gtbd* with quinones prior to induction of catalytic oxygen reduction activity enhances the turnover of the enzyme (Sakamoto et al., 1999; Kusumoto et al., 2000). The activity enhancement observed in these cases may be explained by the binding of quinones to site 2, the putative regulatory or acceleratory quinone binding site. It appears that the access of quinones to site 2 is possible in *Gtbd* and *Cgbd* since no accessory subunits are found at this position in the X-ray and cryoEM structures, respectively. However, Trp^9.A^_*Cgbd/Mtbd*_, which seems important for forming stacking interactions with the naphthoquinone head group of bound MK-9, is not conserved in *Gtbd*. The absence of the Trp residue could explain the different preference of preincubated quinone species observed for *Cgbd* and *Gtbd* (Sakamoto et al., 1999; Kusumoto et al., 2000). The MK-9 that binds to site 2 of *Mtbd* is derived from endogenous sources, and its amount may vary depending on cultivation conditions and sample preparation procedures such as purification methods and the choice of detergents. Future studies are required to characterize site 2 interactions between the protein environment, the heme *b*_595_ group, and exogenously added quinones in greater detail.

In conclusion, the atomic structure of the cytochrome *bd* oxidase from *C. glutamicum* confirms common features among actinobacterial cytochrome *bd* oxidases: (i) the arrangement of three prosthetic heme cofactors in a triangular manner, (ii) the absence of accessory subunits, (iii) the location of the H- and the O-channels, (iv) structural features of a second quinone binding site (site 2). On the other hand, it is also reaffirmed that there are considerable differences between cytochrome *bd* oxidases of Actinobacteria and those of Firmicutes and Proteobacteria. To gain a deeper and more precise understanding of the catalytic mechanisms of the so-far characterized cytochrome *bd* homologs of Actinobacteria, follow-up structural studies of cytochromes *bd* in the presence of substrate quinols or substrate-derived inhibitors are indispensable.

## Materials and methods

### Materials and production of cytochrome *bd* from *C. glutamicum*

Chemicals were purchased from Sigma unless otherwise stated. Cytochrome *bd* from *C. glutamicum* was produced in *C. glutamicum* subsp. *lactofermentum* (ΔctaD/pPC4-cydABCD). This production strain was established as described previously (Kabashima et al., 2009). In brief, the entire cydABDC genes including its authentic promoter region were amplified from genomic DNA of *C. glutamicum* by polymerase chain reaction (PCR) and inserted into the *E. coli-C. glutamicum* shuttle vector, pPC4. The cytochrome *aa*_3_-deficient mutant of *C. glutamicum* (ΔctaD) was transformed with the resulting plasmid, pPC4-cydABDC. Transformants were selected on solid medium containing 50 μg/ml kanamycin and 10 μg/ml chloramphenicol. Individual colonies were cultured in liquid medium for 30 h and the clone with the highest heme *d* content in membrane fractions was selected. Expression of cytochrome *bd* coding genes is induced at late stationary phase, in response to the low oxygen level due to its native promoter. After harvesting, cells were resuspended in 10 mM NaPi buffer at pH 7.0 containing 0.5% (w/v) NaCl. Cell disruption was performed *via* vigorous mixing with glass beads (diameter: 0.18 mm) in a cell disrupting mixer (Bead-Beater, Biospec). Subsequently, low-speed centrifugation (5,000 g, 10 min, 4°C) was carried out to remove undisrupted cells and cell debris. The supernatant was then subjected to ultracentrifugation (100,000 g, 30 min, 4°C). Pelleted membranes were resuspended in 10 mM NaPi buffer at pH 7.0 and used for further protein purification.

### Purification of cytochrome *bd* from *C. glutamicum*

The membrane fraction was resuspended at 10 mg/ml in a first washing buffer [10 mM NaPi (pH 7.4), 0.5 M NaCl, 0.1 mM phenylmethylsulfonyl fluoride (PMSF), 1 mM benzamidine] and stirred for 30 min at 4°C. The suspension was subjected to ultracentrifugation (100,000 g, 20 min, 4°C) and the pelleted membranes were resuspended at 10 mg/ml in a second washing buffer [10 mM NaPi (pH 7.4), 1.5% Na-cholate, 0.5% Na-deoxycholate and 0.1 M NaCl] and stirred for 30 min at 4°C. The membranes were sedimented at 100,000 g for 20 min and the pellet was subsequently resuspended at a concentration of 5 mg/ml in a solubilization buffer [10 mM NaPi (pH 7.4), 10% glycerol, 1% *n*-dodecyl-β-D-maltoside (DDM), 0.1 mM PMSF and 1 mM benzamidine]. Solubilization was carried out for 90 min at 4°C. The solubilized membrane fraction was separated from remaining crude membranes by centrifugation at 100,000 g for 20 min at 4°C.

The supernatant was applied to a Q-Sepharose column, washed with a buffer containing 10 mM NaPi (pH 7.4), 10% glycerol, 0.05% DDM, 0.1 mM PMSF and 1 mM benzamidine and eluted with a NaCl step gradient (150, 280 and 500 mM NaCl). After desalting of the oxidase containing fractions with an Amicon Ultra-100 K concentrator, the oxidase sample was applied to a DEAE-Toyopearl (Tosoh Bioscience) column, washed with a buffer containing 10 mM NaPi, (pH 7.4), 10% glycerol, 0.05% 2,2-didecylpropane-1,3-bis-β-d-maltopyranoside (LMNG), 0.1 mM PMSF and 1 mM benzamidine and eluted by a NaCl step gradient (150, 280 and 500 mM NaCl). Cytochrome *bd* elutes at a NaCl concentration of 280 mM from the DEAE-Toyopearl column. The oxidase containing fractions were pooled, flash-frozen in liquid nitrogen and stored at −80°C until further use.

For sample polishing, the sample was thawed on ice, subjected to centrifugation (21,130 g, 10 min, 4°C) and applied to a Superdex™ 200 Increase 10/300 GL column (GE Healthcare), which had been pre-equilibrated with 3 CV of a buffer containing 20 mM Tris/HCl (pH 7), 100 mM NaCl and 0.002% (w/v) LMNG. The chromatography was performed on an Äkta™ pure system (GE Healthcare) with a flow rate of 0.5 ml/min and a fraction volume of 0.2 ml. Peak fractions were pooled and concentrated to 2.5 mg/ml for downstream cryoEM sample preparation.

### Single-particle cryoEM sample vitrification and data acquisition

#### Sample vitrification

Quantifoil R1.2/1.3 300 mesh Au-carbon grids were washed in chloroform and subsequently glow discharged with a PELCO easiGlow device at 15 mA for 90 s. A volume of 4 μL sample was applied on a grid immediately before plunge freezing. Samples were vitrified at 4°C, 100% humidity, and a blot force of 20 using a Vitrobot IV device (Thermo Scientific). Blotting was carried out for 4 s before plunge-freezing in liquid ethane.

#### Data collection

Electron micrographs were recorded using a Titan Krios G3i microscope operated at 300 kV (Thermo Scientific). Data were acquired automatically using EPU software (Thermo Scientifc) with aberration free image shift (AFIS) in faster acquisition mode using a K3 direct electron detector in combination with a post-column energy-imaging filter (Gatan) at a nominal magnification of ×105,000, corresponding to a calibrated pixel size of 0.837 Å. Dose-fractionated movies were recorded for 4.99 s at an electron flux of 15 e^−^ x pixel^−1^ x s^−1^, corresponding to a total dose of about 107 e^−^ x A^−2^. A focus range of −1.1 to −2.1 μm was applied throughout data collection.

### Data processing

Movies were motion corrected and dose weighted with MotionCor2 using a 5 × 5 patch (Zheng et al., 2017). Initial CTF (contrast transfer function) parameters for each movie were determined with Gctf (Zhang, 2016). At this step images with estimated poor resolution (>3.2 Å) and high astigmatism (>250) were removed. Autopicking was performed using crYOLO (Wagner et al., 2019) and all further data processing including 2D classification, 3D classification, 3D refinement and Bayesian polishing was performed using Relion 3.1 (Zivanov et al., 2018). A total of 10,586,211 particles were picked and cleaned up with four rounds of two-dimensional (2D) classification. One round of 3D classification provided the final particle stack of 410,010 particles which was used for all subsequent rounds of auto-refinement, CTF refinement and Bayesian polishing. Final maps were generated after subtraction of the detergent micelle followed by CTF refinement. An overview of the processing workflow is given in Supplementary Figure S2.

### Model building and map validation

All model-building steps were performed using COOT (version 0.9.6) (Emsley et al., 2010). The cryoEM structure of cytochrome *bd* from *Mycobacterium tuberculosis* (pdb:7NKZ) was used as starting model (Safarian et al., 2021). After backbone fitting and side chain docking, we performed real-space refinement in Phenix (version 1.18.2). Refinement results were manually inspected and corrected if necessary. Map-to-model cross validation was performed in Phenix (version 1.18.2). FSC_0.5_ was used as cut-off to define resolution. Model parameters and corresponding cryoEM map statistics are summarized in Supplementary Table S1. The finalized model was visualized using ChimeraX (Goddard et al., 2018). Tunnels and interior cavities were mapped with MOLE2.5 (bottleneck radius: 1.1 Å, bottleneck tolerance: 3 Å, origin radius 5 Å, surface radius 8 Å, probe radius 5 Å, interior threshold 1.1 Å) (Sehnal et al., 2013; Pravda et al., 2018).

## Data Availability

The cryoEM map file of cytochrome *bd* from *C. glutamicum* was deposited in the EMD database and can be found under accession number EMD-15851. The model file of cytochrome *bd* from *C. glutamicum* was deposited to the PDB under accession number 8B4O.
