# Supplementary material for: The cryoEM structure of cytochrome bd from C. glutamicum provides novel insights into structural properties of actinobacterial terminal oxidases
Source: Front Chem. 2023 Jan 4;10:1085463. doi: 10.3389/fchem.2022.1085463 (PMC9846854; doi:10.3389/fchem.2022.1085463)
Supplement: Supplementary file 1 [file DataSheet1.docx]

Fig. S1.


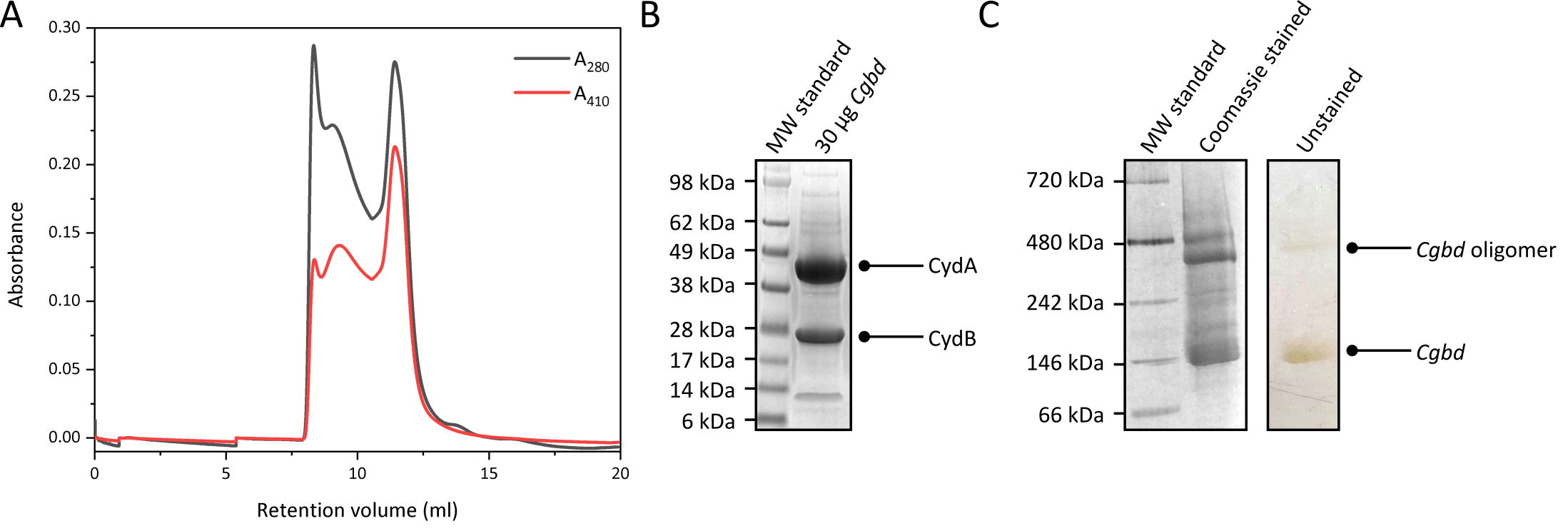


**Fig. S1. Purification of cytochrome *bd* from *C. glutamicum*. (A)** SEC elution profile, **(B)** SDS-PAGE and **(C)** native PAGE of purified *Cgbd.*

Fig. S2.


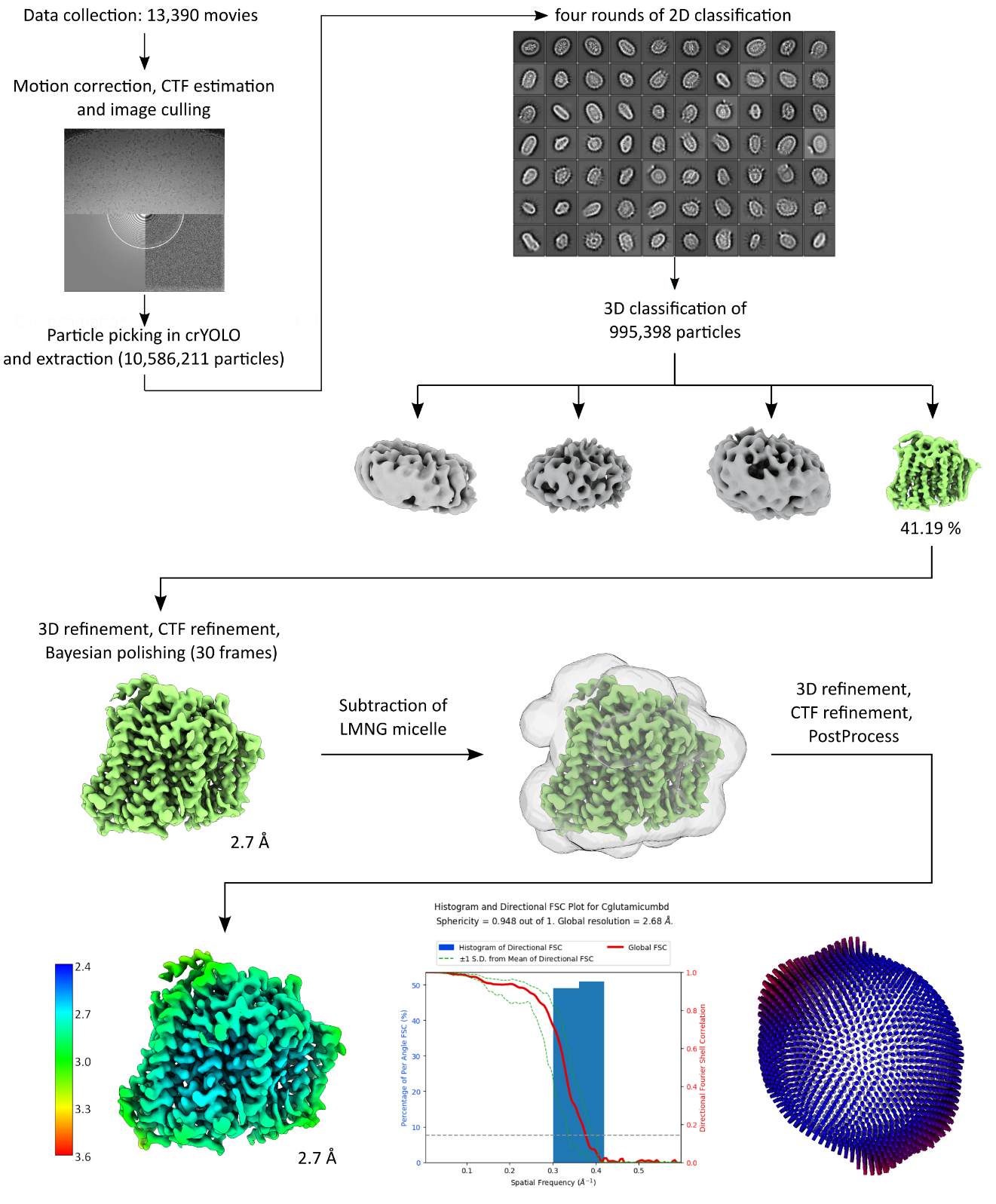


**Fig. S2. CryoEM data processing scheme.** Statistical data are summarized in Supplementary Table 1. Unless otherwise stated, processing was performed with RELION3.1 [(Zivanov et al., 2018)](https://sciwheel.com/work/citation?ids=6071735&pre=&suf=&sa=0&dbf=0). The final map is depicted as local resolution map with corresponding 3DFSC [(Tan et al., 2017)](https://sciwheel.com/work/citation?ids=3906518&pre=&suf=&sa=0&dbf=0) plot and Euler angle distributions. For further details see Material and Methods. Reported resolution values correspond to FSC = 0.143.

**Fig. S3.**


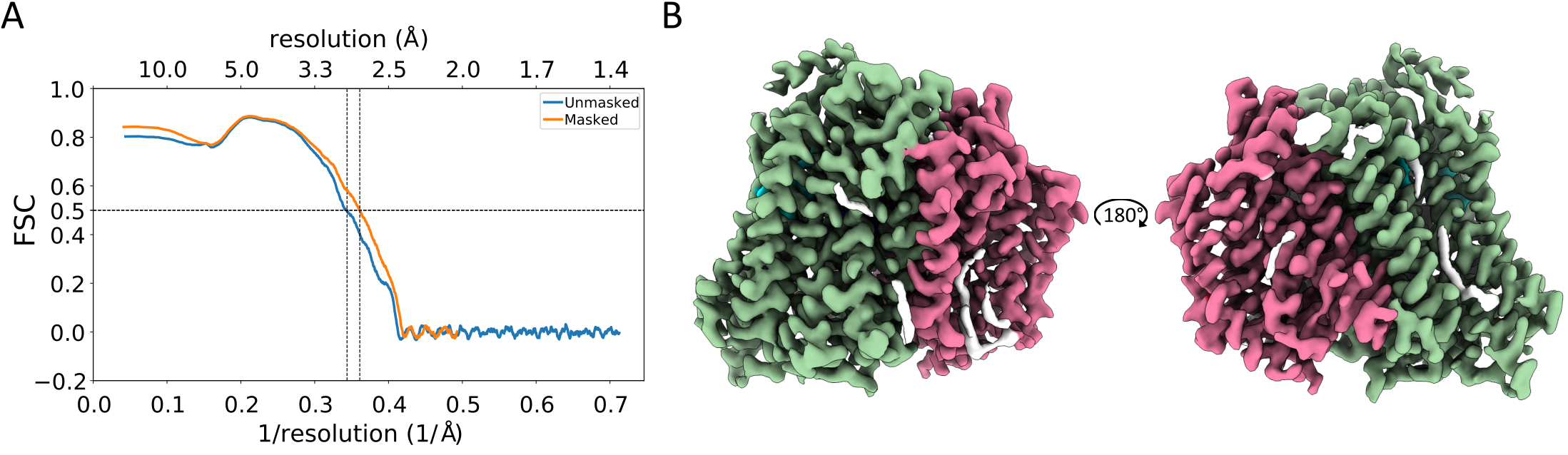


**Fig. S3. Map-to-model correlation. (A)** Map-to-model correlation curve. **(B)** CryoEM density map of *Cgbd*. CydA is colored in green, and CydB in pink. Densities that were uninterpretable are colored in white.

Fig. S4.


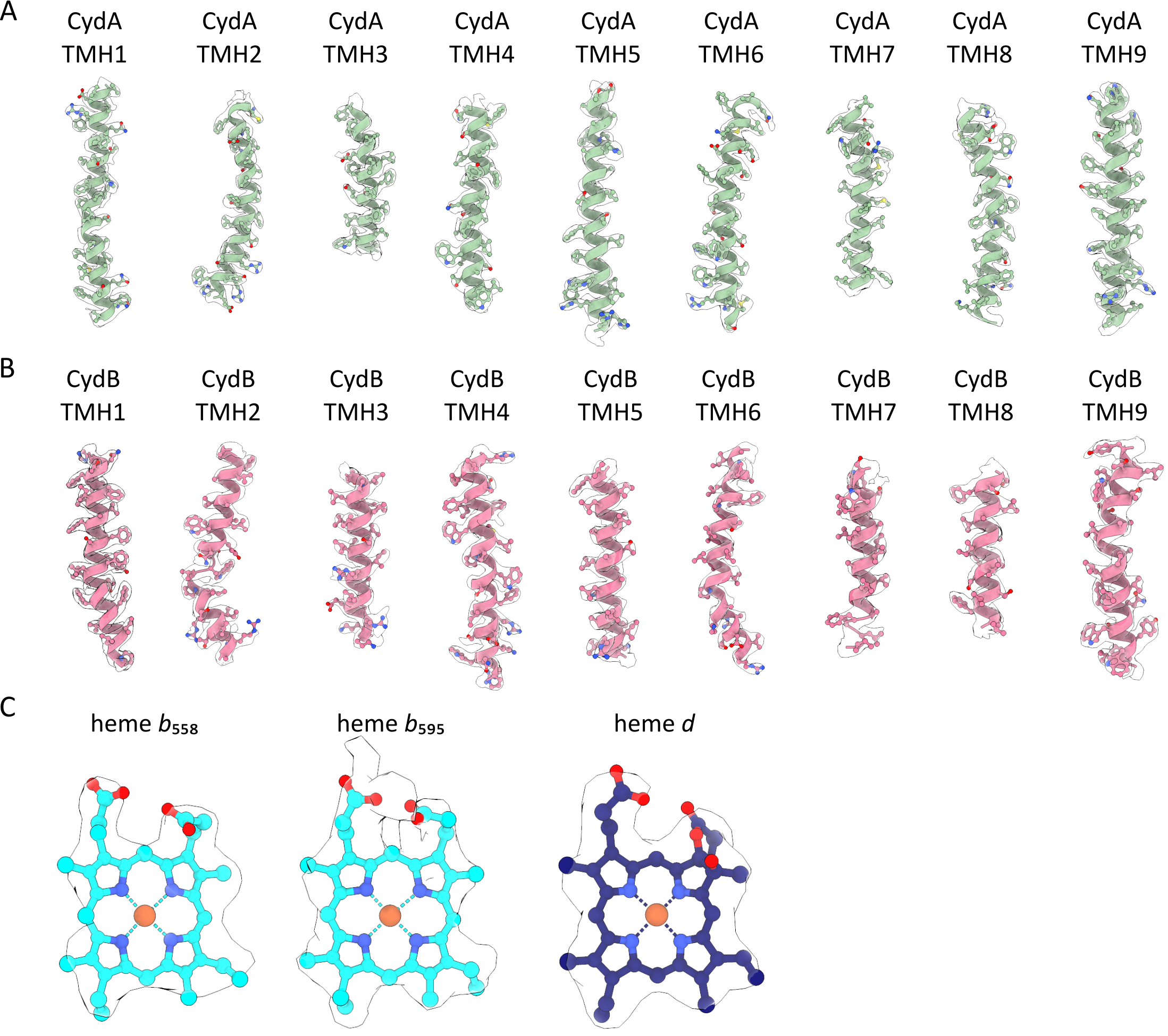


**Fig. S4. Density features of representative structural segments**. Density features of transmembrane helices of **(A)** CydA, and **(B)** CydB; and **(C)** cofactors (heme *b*_558_, heme *b*_595_ and heme *d*). Shown densities are sharpened with a b-factor of -10.


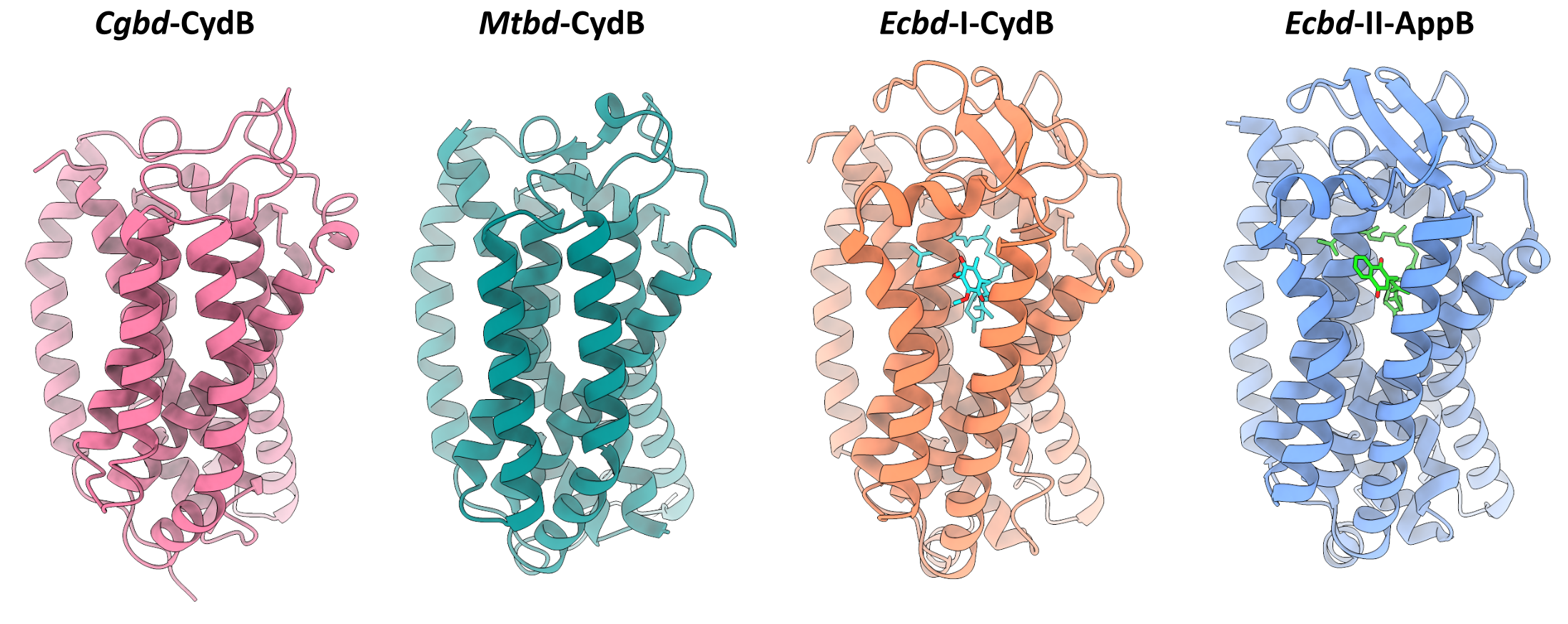


**Fig. S5. Comparison of the linker between TMHs 6 and 7 of the second subunit in cytochrome *bd* structures.** TMHs 6 and 7 of the actinobacterial enzymes (*Mtbd*: 7NKZ) [(Safarian et al., 2021)](https://sciwheel.com/work/citation?ids=11642048&pre=&suf=&sa=0&dbf=0) are linked by a short stretch of amino acids. The *E. coli* oxidases (*Ecbd*-I: 6RKO; *Ecbd*-II: 7OY2) [(Safarian et al., 2019; Grund et al., 2021)](https://sciwheel.com/work/citation?ids=7586482,12904580&pre=&pre=&suf=&suf=&sa=0,0&dbf=0&dbf=0) contain a β-sheet followed by a short α-helix resulting in a bend of TMH7 to allow for space for the structural quinone molecule.


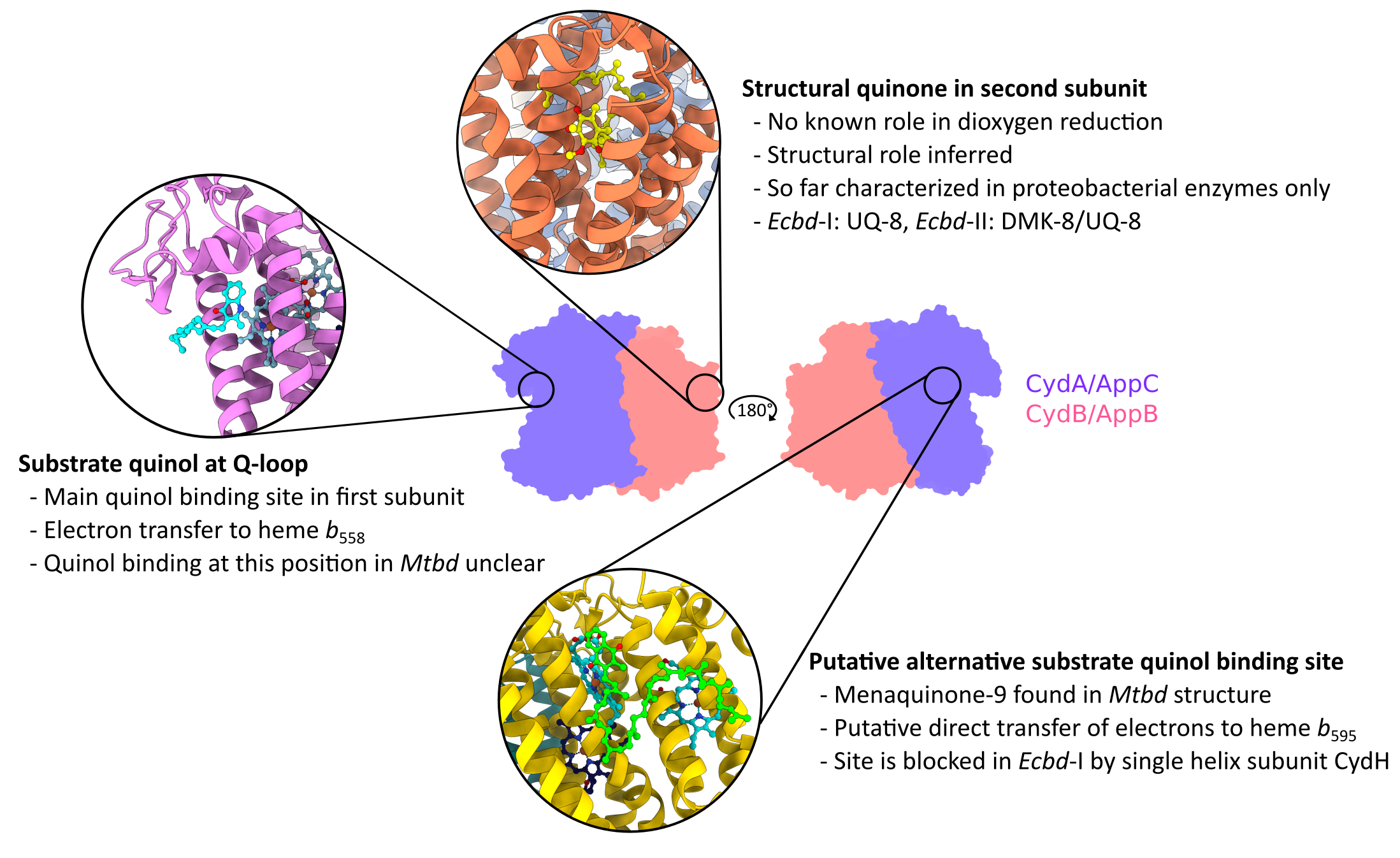


**Fig. S6. Overview and comparison of the quinol/quinone binding sites in cytochromes *bd*.** Three quinol/quinone binding sites have been characterized in structurally characterized cytochromes *bd*. Depicted are the structures of *Ecbd*-I, *Ecbd*-II and *Mtbd* [(Safarian et al., 2016, 2021; Grauel et al., 2021)](https://sciwheel.com/work/citation?ids=1449671,11642048,12783545&pre=&pre=&pre=&suf=&suf=&suf=&sa=0,0,0&dbf=0&dbf=0&dbf=0).

**Supplementary Table 1. CryoEM data collection and validation.** CryoEM data statistics of *as isolated C. glutamicum* *bd* oxidase.

|  | *as isolated* |
| --- | --- |
| **Data collection** |  |
| Accession number | EMD-15851 |
| Magnification | 105k |
| Voltage / kV | 300 |
| Dose / e^-^Å^-2^ | 107 |
| Pixel size / Å | 0.837 |
| Defocus range / µm | -1.1. to -2.1 |
| Recorded movies | 13,390 |
| Final particle images | 410,010 |
| Camera | Gatan K3 |
| Microscope | Titan Krios G3i |
| Energy Filter | BioQuantum K3 |
|  |  |
| **Image processing** |  |
| Initial model |  |
| Resolution (FSC_0.143_) / Å | 2.71 |
| Applied B-factor / Å^2^ | -10 |
|  |  |
| **Model refinement** |  |
| PDB accession | 8B4O |
| Validation |  |
| FSC^map-to-model^_(0.5)_ / Å | 2.77 |
| MolProbity score | 1.76 |
| Composition |  |
| Atoms | 6156 |
| Protein residues | 772 |
| Waters | 0 |
| Ligands | 2 HEB, 1 HDD |
| Bonds (R.M.S.D.) |  |
| Length (Å) | 0.007 |
| Angles (°) | 1.112 |
| B-factors (min/max/mean) |  |
| Protein | 14.62/72.40/32.71 |
| Ligand | 16.06/29.65/23.64 |
| Waters | - |
| Clash score | 17.45 |
| Ramachandran plot (%) |  |
| Favored | 97.91 |
| Allowed | 2.09 |
| Outliers | 0 |
| Rotamer outliers (%) | 0.00 |

Supplementary references

[Grauel, A., Kägi, J., Rasmussen, T., Makarchuk, I., Oppermann, S., Moumbock, A. F. A., Wohlwend, D., Müller, R., Melin, F., Günther, S., et al. (2021). Structure of Escherichia coli cytochrome bd-II type oxidase with bound aurachin D. *Nat. Commun.* 12, 6498. doi:10.1038/s41467-021-26835-2.](https://sciwheel.com/work/bibliography/12783545)

[Grund, T. N., Radloff, M., Wu, D., Goojani, H. G., Witte, L. F., Jösting, W., Buschmann, S., Müller, H., Elamri, I., Welsch, S., et al. (2021). Mechanistic and structural diversity between cytochrome bd isoforms of Escherichia coli. *Proc Natl Acad Sci USA* 118. doi:10.1073/pnas.2114013118.](https://sciwheel.com/work/bibliography/12904580)

[Safarian, S., Hahn, A., Mills, D. J., Radloff, M., Eisinger, M. L., Nikolaev, A., Meier-Credo, J., Melin, F., Miyoshi, H., Gennis, R. B., et al. (2019). Active site rearrangement and structural divergence in prokaryotic respiratory oxidases. *Science* 366, 100–104. doi:10.1126/science.aay0967.](https://sciwheel.com/work/bibliography/7586482)

[Safarian, S., Opel-Reading, H. K., Wu, D., Mehdipour, A. R., Hards, K., Harold, L. K., Radloff, M., Stewart, I., Welsch, S., Hummer, G., et al. (2021). The cryo-EM structure of the bd oxidase from M. tuberculosis reveals a unique structural framework and enables rational drug design to combat TB. *Nature communications*.](https://sciwheel.com/work/bibliography/11642048)

[Safarian, S., Rajendran, C., Müller, H., Preu, J., Langer, J. D., Ovchinnikov, S., Hirose, T., Kusumoto, T., Sakamoto, J., and Michel, H. (2016). Structure of a bd oxidase indicates similar mechanisms for membrane-integrated oxygen reductases. *Science* 352, 583–586. doi:10.1126/science.aaf2477.](https://sciwheel.com/work/bibliography/1449671)

[Tan, Y. Z., Baldwin, P. R., Davis, J. H., Williamson, J. R., Potter, C. S., Carragher, B., and Lyumkis, D. (2017). Addressing preferred specimen orientation in single-particle cryo-EM through tilting. *Nat. Methods* 14, 793–796. doi:10.1038/nmeth.4347.](https://sciwheel.com/work/bibliography/3906518)

[Zivanov, J., Nakane, T., Forsberg, B. O., Kimanius, D., Hagen, W. J., Lindahl, E., and Scheres, S. H. (2018). New tools for automated high-resolution cryo-EM structure determination in RELION-3. *eLife* 7. doi:10.7554/eLife.42166.](https://sciwheel.com/work/bibliography/6071735)
